# Lumbar disc herniation in osteogenesis imperfecta associated with a COL1A1 frameshift mutation: A case report and review

**DOI:** 10.1097/MD.0000000000043451

**Published:** 2025-08-01

**Authors:** Wugui Chen, Guangfeng Ling, Hengmei Chen, Shijie Chen, Jiaqing Huang, Chengshou Lin, Chengzhao Liu

**Affiliations:** a Department of Spinal Surgery, Fujian Medical University Affiliated Mindong Hospital, Ningde, Fujian Province, China.

**Keywords:** AlphaFold 3, case reports, COL1A1, lumbar disc herniation, osteogenesis imperfecta

## Abstract

**Rationale:**

Osteogenesis imperfecta (OI) is a genetic disorder of mesenchymal hypoplasia and collagen defects. Whether abnormal type I collagen predisposes OI patients to multilevel lumbar disc herniation remains unclear.

**Patient concerns:**

An 18-year-old male with childhood-diagnosed type I OI (fragility fractures, blue sclera, dentinogenesis imperfecta, severe osteoporosis) developed progressive low-back pain and bilateral radiculopathy.

**Diagnoses:**

Magnetic resonance imaging revealed multilevel lumbar disc herniation with relatively mild nucleus pulposus degeneration. Whole-exome sequencing identified a de novo COL1A1 frameshift mutation (c.441delC, p.Gly148Aspfs*117) resulting in premature termination. AlphaFold 3 modelling predicted markedly truncated and structurally altered chains.

**Interventions:**

Minimally invasive microdiscectomy, systemic antiosteoporosis therapy (bisphosphonate, calcium/vitamin D), and staged functional rehabilitation were implemented.

**Outcomes:**

Neurological symptoms improved postoperatively during >2 years of follow-up, while a new femoral fracture occurred in 2023.

**Lessons:**

OI patients with COL1A1/COL1A2 mutations may have heightened susceptibility to disc herniation despite modest disc degeneration. Integrating magnetic resonance imaging, genetic testing, and artificial intelligence structural modelling refines diagnosis and pathophysiological understanding. Multidisciplinary management combining targeted surgery, antiosteoporosis therapy, and rehabilitation optimizes long-term outcomes.

## 1. Introduction

Osteogenesis imperfecta (OI), or brittle bone disease, has a prevalence of 0.008% to 0.009% and exhibits highly variable clinical presentations. Characteristic symptoms include fragility fractures from minor trauma, along with features such as short stature, blue sclerae, deafness, bleeding tendencies, indented skull base, joint laxity, obstructive pulmonary disease, and cardiovascular issues. OI, which stems from mesenchymal tissue hypoplasia and collagen formation defects, has an etiology that is not yet fully understood.^[1]^ The spine is a major site affected by OI, with reported complications such as scoliosis and lumbar instability.^[2]^ However, co-occurrence of OI with lumbar intervertebral disc herniation is relatively rare, with limited reports and treatment experience.^[3]^ This report details a case of OI with multilevel lumbar disc herniation, highlighting its diagnostic and therapeutic journey.

## 2. Case reports

**Figure 1.**
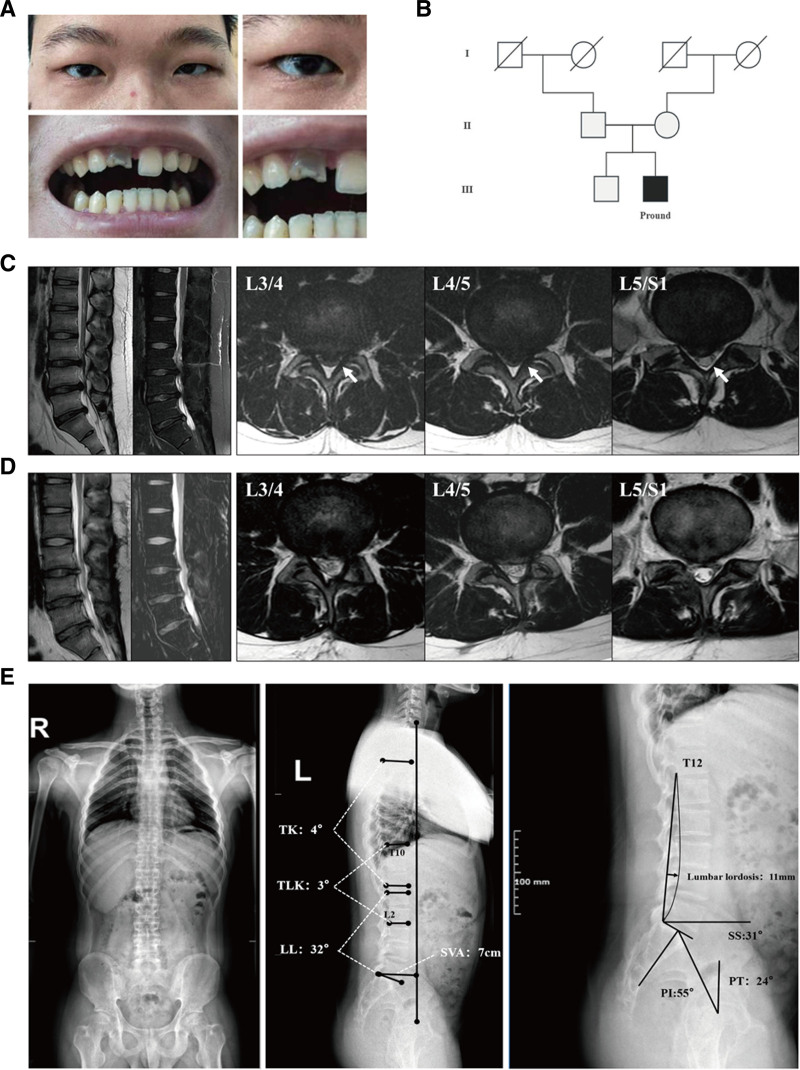
Clinical and radiological findings in an OI patient with intervertebral disc herniation. (A) Blue discoloration of the sclera and dentin dysplasia in the OI patient. (B) Family pedigree map showing the characteristics of OI in the patient’s family. (C) MRI of the lumbar spine before surgery, revealing multilevel intervertebral disc herniation. (D) MRI of the lumbar spine 6 mo after surgery, demonstrated significant improvements. (E) Full-length radiograph of the spine and pelvic parameters, indicating significant changes in spinal alignment. LL = lumbar lordosis, MRI = magnetic resonance imaging, OI = osteogenesis imperfecta, PI = pelvic incidence, PT = pelvic tilt, SS = sacral slope, SVA = sagittal vertical axis, TLK = thoracolumbar kyphosis, TK = thoracic kyphosis.

An 18-year-old male college student, 173 cm tall and weighing 88 kg, with a magnetic resonance imaging of 29.4, and presented with a normal mental state and intelligence. He exhibited blue sclerae, hypoplastic teeth and symptoms consistent with OI (Fig. 1A).^[1]^ His family comprised his parents and brother, neither of whom had a history of similar cases or other genetic diseases (Fig. 1B).

In January 2022, the patient presented to our hospital complaining of lumbago and left lower limb numbness. Magnetic resonance imaging revealed intervertebral disc herniations at L3-4, L4-5, and L5-S1 with left lateral nerve compression, leading to an initial diagnosis of lumbar intervertebral disc herniation (Fig. 1C). After conservative treatment failed to provide relief until March 2022, the patient underwent posterior lumbar fenestration at L3/4, L4, and L5/S1 at another hospital. However, 6 months post-surgery, the patient still experienced mild lumbago and left lower limb numbness, prompting a return to our hospital.

Full-length spine radiography showed spinal imbalance with.a significant reduction in thoracic kyphosis (TK) and lumbar lordosis (LL). The spinal and pelvic parameters were as follows (Fig. 1E), TK (T4-12): 4°; thoracolumbar kyphosis (T10-L2), 3°; (LL; L1-S1), 32°; LL depth, 11 mm; sagittal vertical axis, 7 cm; pelvic incidence, 55°; pelvic tilt, 24°; and sacral slope, 31°. Bone density examination revealed severe osteoporosis with a lumbar spine *Z*-score value of −3.4 and a femoral neck *Z*-score value of −2.0. Postoperative magnetic resonance imaging showed significant improvement in intervertebral disc herniation, spinal canal stenosis, and nerve compression (Fig. 1D). Intervertebral disc degeneration was classified as grade II according to the Pfirrmann classification.

The patient had experienced at least 4 severe fragility fractures since birth, including 3 femoral and 1 humeral fractures, which had been managed with conservative or surgical treatment. At age 12 years, he began a 4-year course of oral alendronate sodium therapy. The most recent femoral fracture in June 2023 required surgery at our hospital, but did not heal even 4 months after surgery. We then recommended calcium and alendronate therapy, as suggested in the literature,^[4]^ leading to significant fracture healing 7 months post-surgery.

### 2.1. Genetic testing and protein modeling

**Figure 2.**
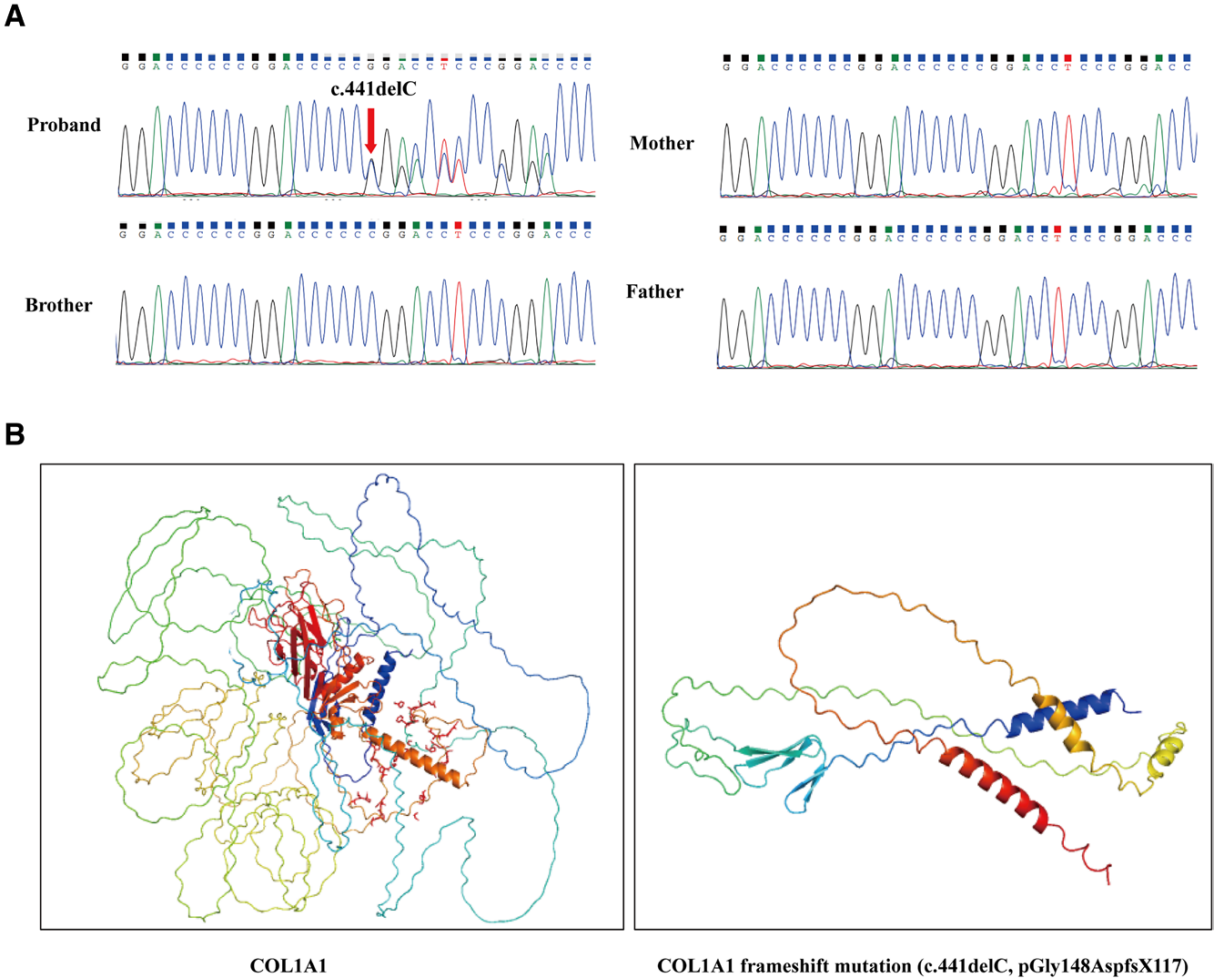
Genetic testing and protein modeling in an OI patient with intervertebral disc herniation. (A) Whole-exome sequencing revealed a sporadic COL1A1 mutation (pGly148Aspfs*117). (B) The mutated protein structure was predicted using AlphaFold3 modeling, demonstrating significant shortening and structural variation compared to the wild type COL1A1 protein. COL1A1 = collagen type I alpha 1, OI = osteogenesis imperfecta.

To further clarify the genetic characteristics and phenotypes of this patient, we performed whole-exome sequencing tests after obtaining consent from the patient and his family. Whole-exome sequencing of the 4 family members revealed a sporadic collagen type I alpha 1 (COL1A1) mutation (pGly148Aspfs*117; Fig. 2A), indicating that at amino acid 148 of the protein, the original glycine was replaced by aspartate because of the frameshift mutation, resulting in a subsequent alteration of the amino acid sequence and the emergence of a stop codon at the 117th amino acid, which affects the synthesis and function of COL1A1. To predict the protein structure, we used AlphaFold3 modeling (https://alphafoldserver.com/),^[5]^ as shown in Figure 2B, which demonstrates a significantly shortened protein sequence and structural variation compared to the original COL1A1 structure.

### 2.2. Treatment and follow-up

Following conservative treatment with alendronate sodium, mecobalamin, tizanidine, and rehabilitation guidance that included lumbar back muscle exercises and weight control, the patient’s symptoms significantly improved. To date, he has been under follow-up for more than 2 years.

## 3. Discussion

OI is a genetic skeletal dysplasia characterized by enhanced bone fragility and deformities. Its complex phenotype leads to its diagnosis, primarily through clinical symptoms and radiography. Key diagnostic criteria included a history of childhood fragility fractures, blue sclerae, hearing loss, familial fracture history, and characteristic bone radiograph features. Genetic testing and biochemical markers can further assist in diagnosis. Professor David Sillence’s 1979 classification^[6]^ divides OI into 4 types, based on genetics and clinical presentation. Types II and III exhibit severe symptoms and high mortality, whereas types I and IV show milder symptoms and higher survival rates.^[7]^ Our patient, with blue sclera, dentin hypoplasia, and multiple fragility fractures, was diagnosed as Type IB OI.

OI is primarily a genetic disorder of the connective tissue due to mutations in the genes encoding type I collagen (COL1A1 and COL1A2), which account for approximately 90% of cases.^[8]^ Type I collagen and proteoglycans are key components of the intervertebral disc framework, and are crucial for maintaining their shape and tension. The annulus fibrosus of the intervertebral disc is mainly composed of type I collagen fibers, whereas the nucleus pulposus primarily composed of type II collagen fibers, with compositional and structural changes occurring as degeneration progresses.^[9]^ Consequently, OI patients with abnormal collagen synthesis may face an increased risk of lumbar disc herniation, a rarely reported condition. Peng et al described a 19-year-old male OI patient with L4/5 lumbar disc herniation who recovered satisfactorily after percutaneous endoscopic discectomy.^[10]^ Wang et al reported a 32-year-old OI patient with Schmorl’s disease and a COL1A2 heterozygous mutation (c.4048G>A).^[3]^, exhibiting multiple vertebral fragility fractures and Schmorl nodules. In our case, despite relatively mild disc degeneration (Pfirrmann grade II), the patient presented with severe multilevel disc herniation and nerve compression. Therefore, we speculate that type I collagen abnormalities might weaken the annulus fibrosus, contributing to disc herniation in young OI patients.

To validate this hypothesis, whole-exome sequencing was conducted on the patient’s family, which revealed a de novo mutation in COL1A1 (c.441delC, p.Gly148Aspfs*117). This mutation site was previously reported by Zhang^[11]^ and Lin^[12]^ in Chinese individuals with type I OI, although neither study has discussed intervertebral disc herniation phenotypes. Using AlphaFold3 modeling, the mutated COL1A1 protein was reconstructed, indicating significant protein sequence shortening and structural changes. Such technological advancements offer enhanced insights into the correlation between protein mutations and disease mechanisms.^[5]^

To investigate the genetic basis of this unique case, whole-exome sequencing was performed on the patient’s family, revealing a denovo mutation in COL1A1 (c.441delC, p.Gly148Aspfs*117). This mutation site was previously reported by Zhang and Lin in Chinese individuals with type I OI, but neither study mentioned intervertebral disc herniation phenotypes. Using AlphaFold3 modeling, the mutated COL1A1 protein was reconstructed, showing significant protein sequence shortening and structural changes. While the frameshift mutation theoretically results in a truncated protein, the actual production of this protein is questionable due to nonsense-mediated decay, especially as the mutation is not in the last exon of the gene. However, the 3-dimensional modeling results were presented to highlight the theoretical differences in protein synthesis and to provide a visual reference for understanding the potential impact of the mutation. This approach not only illustrates the theoretical truncation but also offers a valuable perspective for studying the structural implications of similar mutations in future research. Such technological advancements enhance our insights into the correlation between protein mutations and disease mechanisms.

In addition to genetic factors affecting collagen, OI patients face an increased risk of disc herniation due to osteoporosis and muscle weakness, thereby can disrupt spinal-pelvic alignment. This disruption increases the mechanical stress on the lumbar intervertebral discs. Numerous previous studies have confirmed that sagittal parameters of the spine pelvis are closely related to the degeneration and clinical manifestations of intervertebral discs.^[13]^ In this case, (TK, 4°), thoracolumbar kyphosis (3°), (LL, 32°), and LL depth (11 mm) were significantly reduced, while the sagittal vertical axis (7 cm) and pelvic tilt (24°) were significantly increased. This indicates a decrease in TK and LL, resulting in increased load bearing on the lower lumbar intervertebral discs and an elevated risk of developing intervertebral disc herniation.

OI, an incurable genetic disorder, is managed through a multidisciplinary approach involving physical therapy, surgery, and bone-targeted drug therapy. The primary goals are to improve bone health, enhance quality of life, and prevent long-term complications. Bisphosphonates increase bone mineral density and reduce fracture rates in OI patients^[14]^ but are less effective than osteoporosis.^[1,15]^ The patient benefited from over 4 years of alendronate sodium treatment, which ceased at the age of 16. Recently, after femoral fracture nonunion, short-term alendronate use effectively reduced back pain and aided fracture recovery,^[4]^ although the optimal treatment timing and duration remain debated.^[14,15]^ New treatments such as denosumab, teriparatide, TGF-β antibodies, and growth hormone are under research,^[15,16]^ with the off-label use of denosumab requiring careful evaluation.^[17]^

Surgical interventions for OI patients mainly target fracture prevention and management.^[18,19]^ This case is unique because of multilevel disc herniation with nerve compression, a rare occurrence in OI. For young patients with lumbar disc herniation, conservative treatment is typically preferred, with minimally invasive disc resection and lumbar fusion reserved for severe cases.^[20]^ Considering the patient’s age, symptoms, and treatment options, minimally invasive lumbar fenestrated discectomy was selected to achieve satisfactory short-term results. Given the significant osteoporosis and reduced muscle strength common in these patients, comprehensive treatment including muscle strengthening and anti-osteoporosis measures is crucial for symptom relief. In cases of recurrence, lumbar fusion and internal fixation may be required, but the abnormal pedicle development and the risk of internal fixation failure or nonunion due to osteoporosis require more attention.^[18]^ In summary, the main treatment goals for these patients are to alleviate symptoms and improve quality of life through conservative therapies.

In conclusion, we report a novel case of an 18-year-old male OI patient with multilevel lumbar disc herniation caused by a sporadic COL1A1 mutation. This case highlights the crucial role of genetic factors in OI-related complications and underscores the importance of considering nonskeletal manifestations like disc herniation in OI patients. Our comprehensive approach combining minimally invasive surgery, anti-osteoporosis therapy, and functional exercise offers new insights into the management of such complex cases. This case expands our understanding of OI-related complications and highlights the importance of musculoskeletal issues in OI patients. Clinicians should actively evaluate and manage potential spinal problems in OI patients to enhance their quality of life. Future research should focus on confirming the pathogenic mechanisms linking OI and disc herniation, collecting and analyzing clinical specimens from OI patients, and exploring new therapeutic approaches such as advanced genetic therapies and more effective medications.

## Acknowledgments

The authors would like to express their thanks to Mrs. Xiushan Feng for his professional assistance. Whole-exon testing was provided by Beijing Genomics Institute.

## Author contributions

**Conceptualization:** Wugui Chen, Shijie Chen, Chengshou Lin, Chengzhao Liu.

**Data curation:** Wugui Chen, Guangfeng Ling, Hengmei Chen, Shijie Chen, Jiaqing Huang.

**Funding acquisition:** Wugui Chen, Guangfeng Ling, Chengshou Lin, Chengzhao Liu.

**Investigation:** Guangfeng Ling, Hengmei Chen, Shijie Chen, Jiaqing Huang.

**Methodology:** Shijie Chen, Jiaqing Huang.

**Project administration:** Wugui Chen, Chengshou Lin, Chengzhao Liu.

**Validation:** Hengmei Chen.

**Writing – original draft:** Wugui Chen, Guangfeng Ling, Hengmei Chen, Chengshou Lin.

**Writing – review & editing:** Wugui Chen, Chengshou Lin, Chengzhao Liu.
